# Association with menopausal hormone therapy and asymptomatic gallstones in US women in the third National Health and Nutrition Examination Study

**DOI:** 10.1038/s41598-023-50509-2

**Published:** 2024-01-02

**Authors:** Sarah S. Jackson, Barry I. Graubard, Chiara Gabbi, Jill Koshiol

**Affiliations:** 1 Division of Cancer Epidemiology and Genetics, Infections and Immunoepidemiology Branch, National Cancer Institute, 9609 Medical Center Drive, Bethesda, MD 20879 USA; 2 Department of Biosciences and Nutrition, Karolinska Institutet, Stockholm, Sweden

**Keywords:** Gastroenterology, Epidemiology

## Abstract

15% of US adults have gallstones, most of which are clinically “silent”. Several studies show that menopausal hormone therapy (MHT) increases symptomatic gallstones and cholecystectomy risk. MHT use may be contraindicated in women with gallstones and population studies may be biased by “confounding by contraindication” while the true association between MHT and gallstones remains underestimated. We sought to examine whether MHT use was associated with asymptomatic gallstones using instrumental variable (IV) analysis to account for confounding by contraindication. We used 2018 postmenopausal women from the Third National Health and Nutrition Examination Survey to estimate associations of MHT use with asymptomatic gallstones. A traditional logistic regression analysis was compared to instrumental variable (IV) analysis to account for confounding by contraindication. 12% of women with asymptomatic gallstones and 25% of women without gallstones were current MHT users (*P* < 0.001). The traditional analysis suggested a decreased odds of asymptomatic gallstones in current versus never users (OR 0.58, 95% CI 0.37, 0.89), but increased odds (OR 1.51, 95% CI 0.44, 5.16) in the IV analysis. The traditional analysis consistently underestimated the odds of asymptomatic gallstones with MHT use compared to the IV analysis. Accounting for confounding by contraindication, we found a suggestive, though imprecise, positive association between MHT use and asymptomatic gallstones among postmenopausal women. Failure to consider contraindication can produce incorrect results.

## Introduction

Gallbladder disease affects 10–15% adults in the United States (US)^1^. The majority of gallstones are clinically “silent”, and 50–75% of individuals do not experience symptoms like biliary colic, acute cholecystitis, cholangitis, or pancreatitis^1^. Yet, both symptomatic and asymptomatic gallstones are associated with an increase in overall mortality related to cardiovascular disease and cancer^2,3^.

Gallstones are more common among women than men, suggesting that sex steroid hormones play a role in gallstone formation and gallbladder cancer^4,5^. Indeed, estrogens promote the development of gallstones by increasing cholesterol secretion and decreasing bile salt secretion, while progestins reduce gallbladder emptying leading to bile stasis^1^. From the 1970s to 1980s, several observational studies showed that exogenous sources of these sex hormones, such as menopausal hormone therapy (MHT), were associated with gallstones^6^. An elevated risk of self-reported gallstones was noted in the Women’s Health Initiative (WHI)^7^, and an increased incidence of cholecystectomy was seen in the Heart and the Estrogen/progestin Replacement Study (HERS-II)^8^ among women randomized to MHT. MHT use has been associated with both an increased^5^ and a decreased risk of gallbladder cancer^9,10^, for which gallstones are the major risk factor^1^.

Previous analyses of MHT and gallstones that use indicators of symptomatic gallstones, such as diagnosed cholestasis, cholelithiasis, or cholecystectomy, are hindered by detection bias as symptomatic gallstones represent just a small proportion of those with gallstones^11^. While confounding by indication may occur when the indication for treatment is also a risk factor for the outcome (e.g. major depressive disorder among antidepressant users)^12,13^, confounding by contraindication can occur when patients most at risk for the outcome are least likely to be prescribed the treatment under study^12,14^. Clinicians may have been less likely to prescribe MHT to women at risk for gallstones (e.g., due to family history, obesity, high parity, etc.). Therefore, if women least likely to develop gallstones are more often prescribed MHT this may induce an inverse association between MHT use and gallstones and a traditional analysis would be biased. Instrumental variable (IV) analysis is an approach to obtain unbiased treatment effect estimates when there is confounding by indication or contraindication. IV analysis mirrors the treatment assignment of a randomized clinical trial by choosing an instrument that predicts the exposure and has no independent association with the outcome. We sought to examine whether MHT use was associated with asymptomatic gallstones accounting for confounding by contraindication among US women in the Third National Health and Nutritional Examination Survey (NHANES III) comparing an IV analysis to a traditional epidemiologic analysis.

## Methods

The NHANES III is a national probability survey conducted by the US Centers for Disease Control and Prevention in two phases from 1988 to 1994 involving over 60,000 Americans^15^. This cross-sectional survey was designed to be nationally representative of the civilian, non-institutionalized population. The survey consisted of a home interview and a portion of these participants underwent a detailed clinical examination. All participants provided signed informed consent and the study was approved by a human subjects committee within the US Department of Health and Human Services. Data were available for 2941 postmenopausal female participants 20–74 years of age who were interviewed and underwent examination by abdominal ultrasonography. Women were considered post-menopausal if they reported not having a menstrual period in the past 12 months.

### Outcome definition

Ultrasonography of the gallbladder was performed on adults aged 20–74 years by trained technicians using standardized procedures^16^. Participants were asked to fast for at least 6 h prior to the exam but were not excluded if they had not fasted. All examinations were videotaped and reviewed by consulting radiologists specializing in abdominal ultrasonography. Disagreements between radiologists and technicians were adjudicated by a senior radiologist. The level of agreement between the technician’s diagnosis and the radiologist’s diagnosis was found to be excellent^17^.

We were not able to evaluate the use of MHT and symptomatic gallstones in postmenopausal women because NHANES III did not capture age at self-reported gallstone symptoms so the timing of diagnosis relative to MHT initiation is unknown. As such, we excluded women with self-reported symptomatic gallstones if they answered “yes” to either of the following questions on the adult questionnaire: “Has a doctor ever told you that you had gallstones?” (n = 494) or “Have you ever had gallbladder surgery?” (n = 441). We also excluded women with evidence of gallstones on the ultrasound exam who also had abdominal symptoms consistent with biliary colic^18,19^ (pain in the upper right quadrant or epigastric abdomen lasting between 1 and 24 h) (n = 45) or evidence of a cholecystectomy on exam but not reported (n = 18). We further excluded women with missing or inconclusive answers to the above questions or the gallbladder ultrasound exam (n = 202).

Women were classified as having asymptomatic gallstones if they denied a history of gallstones, cholecystectomy, and biliary colic but had evidence of gallstones on the abdominal ultrasound (n = 314). Women who did not report a history of diagnosed gallstones or biliary colic and had a normal gallbladder on the ultrasound examination were considered free of gallbladder disease (n = 1704).

### Measurements

The NHANES III questionnaire was administered by a trained interviewer who collected information on menopausal status and MHT use. Post-menopausal women were asked to report their age at menopause and type of menopause (natural or surgical/medical). Women were asked detailed questions about their use of MHT, including the means of administration (pills, injections, or patches), the age of initiation, duration of use (0–< 1 years, ≥ 1–< 5 years, vs. ≥ 5 years), and time since last use (0–< 1 years, ≥ 1– < 5 years, vs. ≥ 5 years). Women who indicated they were taking MHT at the time of the interview were classified as current users, those indicated they had stopped using MHT were classified as former users, and those who indicated they never used MHT, or their duration of use was < 1 years were never users.

Race/ethnicity was collected by self-report and categorized as White non-Hispanic, Black non-Hispanic, Mexican American, or other. Race/ethnicities in this other category includes Hispanics, regardless of race, who were not Mexican American as well as all non-Hispanics from racial groups other than White or Black, such as individuals of Asian descent^17^. The NHANES III questionnaire also included data on self-reported age, years of education completed, smoking history (current, former, or never), alcohol use (current, former, or never), parity (0, 1–2, 3–4, or ≥ 5 children), use of oral contraceptives, and self-reported history of diabetes and high cholesterol. Body mass index (BMI), calculated in kilograms per squared meters from weight and height measured during the physical examination, was categorized according to the World Health Organization International Classification for Western women as follows: underweight (15.0 to < 18.5 kg/m^2^), normal weight (18.5 to < 25 kg/m^2^), overweight (25 to < 30 kg/m^2^), and obese (≥ 30 kg/m^2^)^20^. Participants were asked if they had taken a non-prescription non-steroidal anti-inflammatory drugs in the past month containing aspirin or ibuprofen. Because statins may be associated with a lower incidence of gallstones, we extracted data on reported statin use in the previous 30 days^21,22^.

### Statistical analysis

We examined the distribution of demographics, medical history, medication use, menopausal variables, and MHT use by gallstone status (asymptomatic gallstones and no gallstones) using sampling weights. To illustrate how the association between MHT use and gallstones can be confounded by contraindication we conducted two analyses. First, we performed a straightforward analysis using logistic regression accounting for the complex, stratified, cluster sampling design with sampling weights to estimate the associations between MHT use and asymptomatic gallstones, adjusting for statistically significant (*P* < 0.05) covariates that included age (continuous), race (White vs. Black/Hispanic/Other), BMI (underweight, normal, overweight, and obese), parity (continuous), age at menopause (continuous), and menopause type (natural vs. surgical). Second, to account for confounding by indication we used IV analysis to examine the association of MHT with asymptomatic gallstones accounting for complex study design and adjusting for the same covariates^23,24^. We chose NHANES geographic region (Northeast, Midwest, South and West) as our instrument after ensuring this variable met the assumptions of a valid instrument (see eMethods in Supplement).

Once our instrument was validated (see Supplement), we used two-stage residual inclusion (2SRI), the binary analogue to two-stage least square regression, to fit IV regression models for estimates of the association between MHT and asymptomatic gallstones^25^. Briefly, this method involves first regressing treatment (MHT) on the IV adjusting for covariates in a logistic regression model. In the second stage, we regressed the outcome (asymptomatic gallstones) on treatment (MHT) with the residuals from the first stage and covariates using logistic regression considering the complex, stratified sampling design with sampling weights and clustering to estimate odds ratios (ORs) and 95% confidence intervals (CI). We examined the associations between MHT use and duration of use with asymptomatic gallstones. Among women with a history of MHT use, we fit models for the association between time since last use and MHT type with asymptomatic gallstones. We compared the beta coefficients between the traditional and IV analyses using a Wald test that included the leaving-one-out jackknife variance estimation of the standard errors and covariances that accounted for both the variability from two step IV and the traditional methods and the complex sample design of NHANES III^26^.

Statistical analyses were conducted in SAS version 9.4 (SAS Institute, Cary, NC) using the SURVEYFREQ, SURVEYMEANS, and SURVEYLOGISTIC procedures to account for the sampling weights and complex survey design.

### Research ethics approval

The NHANES III obtained Institutional Review Board approval through the National Center for Health Statistics and documented consent was obtained from participants (see: https://www.cdc.gov/nchs/nhanes/irba98.htm).

## Results

The characteristics of the postmenopausal women from NHANES III are presented in Table 1 by presence of gallstones. The prevalence of current MHT use was 12% among women with asymptomatic gallstones and 25% among women without gallstones (*P* < 0.001). The average age of women with asymptomatic gallstones was 61.3 years (standard error (SE) = 0.7) and for women without evidence of gallstones was 57.4 years (SE = 0.4, *P* < 0.001). Compared to those without evidence of gallstones, women with asymptomatic gallstones were more likely to be obese (39.4% vs 28.0%, *P* = 0.01), have higher levels of parity (≥ 5 live births 25.2% vs. 16.3, *P* = 0.002), report a history of diabetes (12.3% vs. 8.1%, *P* = 0.02), report an older age at menopause (45.4 [SE = 0.5] years vs. 43.1 years [SE = 0.4], *P* < 0.001), and report natural menopause (67.0% vs. 51.0%, *P* < 0.001). The two groups did not differ in terms of smoking history, education level, past use of oral contraceptives, self-reported high cholesterol, or use of NSAIDs or statins in the past 30 days.

**Table 1:** Selected characteristics of 2407 post-menopausal women by presence of asymptomatic gallstones in NHANES III (1988–1994).

Characteristic	Asymptomatic gallstones N = 314	No gallstones N = 2093	p-values
Demographics			
Age in years, mean (SE)	61.3 (0.7)	57.4 (0.4)	< 0.001
Race/ethnicity, n (%)			0.21
Non-Hispanic White	142 (78.7)	968 (80.3)	
Non-Hispanic Black	69 (9.6)	642 (11.9)	
Mexican American	89 (4.0)	403 (2.8)	
Another race	14 (7.7)	80 (5.0)	
Level of education in years, mean (SE)	11.3 (0.2)	11.7 (0.1)	0.08
BMI categories, n (%)			
Underweight	6 (1.9)	54 (2.6)	0.01
Normal weight	66 (21.0)	645 (30.8)	
Overweight	114 (36.3)	703 (33.6)	
Obese	126 (40.2)	684 (32.7)	
Missing	2 (0.6)	7 (0.3)	
Smoking, n (%)			
Never	176 (50.3)	1140 (48.6)	0.90
Former	76 (25.7)	483 (27.3)	
Current	62 (24.4)	470 (24.2)	
Alcohol use, n (%)			
Never	98 (19.8)	543 (20.3)	0.05
Former	140 (51.1)	965 (43.9)	
Current	76 (29.1)	583 (35.8)	
Missing	0 (0.0)	2 (0.0)	
Parity category, n (%)			
No children	23 (5.2)	269 (13.5)	0.002
1 to 2 children	100 (34.9)	690 (38.1)	
3 to 4 children	91 (34.8)	614 (32.1)	
5 or more children	100 (25.2)	519 (16.3)	
Missing	0 (0.0)	1 (0.0)	
Age at menopause in years, mean (SE)	45.4 (0.5)	43.1 (0.4)	< 0.001
Type of menopause, n (%)			
Natural	211 (67.0)	1066 (51.0)	< 0.001
Surgical	92 (33.0)	776 (49.0)	
MHT use, n (%)			
Never	224 (66.4)	1272 (53.0)	< 0.001
Former	55 (21.7)	405 (21.7)	
Current	35 (12.0)	409 (25.2)	
Ever OC use, n (%)			
Yes	89 (35.1)	771 (41.9)	0.16
No	225 (64.9)	1320 (58.0)	
Missing	0 (0.0)	2 (0.)	
Self-reported diabetes, n (%)			
Yes	57 (12.3)	272 (8.1)	0.02
No	257 (87.7)	1816 (91.9)	
Missing	0 (0.0)	5 (0.0)	
Self-reported high cholesterol, n (%)			
Yes	102 (32.5)	666 (31.8)	0.62
No	109 (34.7)	771 (36.8)	
Missing	37 (11.2)	656 (31.3)	
NSAID use in the past 30 days, n (%)			
Yes	150 (52.5)	964 (46.1)	0.85
No	163 (47.5)	1123 (53.6)	
Missing	1 (0.0)	6 (0.3)	
Statin use in the past 30 days, n (%)			
Yes	23 (7.2)	106 (5.5)	0.29
No	291 (92.8)	1987 (94.5)	

As detected on abdominal ultrasound.

Results are presented accounting for clustering and sampling weights of the complex, multistage survey design.

*BMI* body mass index, *NSAID* non-steroidal anti-inflammatory drug, *n* number, *OC* oral contraceptives, *SE* standard error.

The multivariable associations using a traditional analysis and an IV analysis between MHT use and odds of asymptomatic gallstones are shown in Table 2. In the traditional analysis, current MHT use was associated with a decreased odds of asymptomatic gallstones compared to never use (OR_current_ 0.58, 95% CI 0.37, 0.89). While not statistically significant, in the IV analysis both current and former use had odd ratios for asymptomatic gallstones above 1.0 compared to never users (OR_current_ 1.51, 95% CI 0.44, 5.16; OR_former_ 1.28, 95% CI 0.63, 2.62). Longer duration of MHT use in the traditional analysis was associated with decreased odds of asymptomatic gallstones (OR_≥ 5 years_ 0.50, 95% CI 0.29, 0.86) compared to women with less than one year or no years of use. Conversely, though not statistically significant, the IV analysis suggested that longer duration of MHT use also tended to be associated with an increased odds of asymptomatic gallstones. Women who reported ≥ 5 years of MHT use were 2.08 (95% CI 0.60, 7.17) times as likely to have asymptomatic gallstones than women with 0–< 1 years duration of MHT use. Though not significant, the traditional analysis suggested increasing odds of asymptomatic gallstones with increasing time since last use (OR_≥ 5 years_ 1.52, 95% CI 0.71, 3.23), while the IV analysis suggested time since last use was inversely associated with asymptomatic gallstones, with women who last used MHT ≥ 5 years ago having the lowest odds (OR 0.16, 95% CI 0.03, 0.88). Both the traditional and IV analyses suggested that MHT administration in pill form compared to other administrations, such as creams, injections, or patches, were associated with an increased odds of asymptomatic gallstones (traditional OR 1.43, 95% CI 0.69, 2.98; IV OR 1.40, 95% CI 0.68, 2.89).

**Table 2:** Use of menopausal hormone therapy and odds of asymptomatic gallstones among post-menopausal women in NHANES III (1988–1994).

MHT use	Women with asymptomatic gallstones	Women without gallbladder disease	Traditional analysis OR (95% CI)	IV analysis OR (95% CI)	*P*-value^†^
MHT use					
Never	224	1272	1.00 (Reference)	1.00 (Reference)	0.817
Former	55	405	0.80 (0.50, 1.29)	1.28 (0.63, 2.62)	
Current	35	409	0.58 (0.37, 0.89)	1.51 (0.44, 5.16)	
Duration of use category					
0–< 1 year	246	1450	1.00 (Reference)	1.00 (Reference)	0.738
≥ 1–< 5 years	24	236	0.55 (0.29, 1.03)	1.14 (0.50, 2.62)	
≥ 5 years	31	338	0.50 (0.29, 0.86)	2.08 (0.60, 7.17)	
Among MHT users					
Time since last use category					
0–< 1 years	40	437	1.00 (Reference)	1.00 (Reference)	0.677
≥ 1–< 5 years	11	98	1.06 (0.39, 2.86)	0.37 (0.10, 1.30)	
≥ 5 years	34	249	1.52 (0.71, 3.23)	0.16 (0.03, 0.88)	
MHT type					
Other (creams, injections, or patches)	24	255	1.00 (Reference)	1.00 (Reference)	0.671
Pills	64	538	1.43 (0.69 2.98)	1.40 (0.68, 2.89)	

All models are adjusted for age, race (White vs. Black/Hispanic/Another race), BMI (underweight, normal, overweight, and obese), parity (continuous), age at menopause (continuous), and menopause type (natural vs. surgical), accounting for clustering and sampling weights of the complex, multistage survey design.

As detected on abdominal ultrasound.

*95% CI* 95% confidence interval, *MHT* menopausal hormone therapy, *OR* odds ratio.

^†^*P*-value from a using a Wald test for the differences in the beta coefficients between the traditional and instrumental variable analysis.

When compared statistically, the results from the traditional and IV analyses were not statistically significant different from one another for any of the comparisons (Wald *P* > 0.05). However, the ORs from the traditional and IV methods suggest opposite associations for most comparisons.

## Discussion

We examined the association between MHT use and asymptomatic gallstones identified through abdominal ultrasound among postmenopausal women in a nationally representative survey. Clinicians may be less likely to prescribe MHT to women with strong risk factors for gallstones such as being overweight or obese, with high parity, or family history^1^. As hypothesized, our initial analysis underestimated the association between MHT use and asymptomatic gallstones. Using IV methods to account for confounding by contraindication, though not statistically significant, our results suggest that current users of MHT may be more likely to have asymptomatic gallstones than never users, and that the odds increased with longer duration of use and decreased with longer time since last use. These results also suggest that women taking non-oral forms of MHT may have a reduced odds of asymptomatic gallstones.

Our results should be interpreted with caution as most of these findings did not reach statistical significance. However, our findings are in line with research on MHT use and symptomatic gallstones among postmenopausal women. Several studies found an increase in cholecystectomy with MHT use^27–31^, and others have found similar increases in symptomatic cholelithiasis with MHT use^32,33^. A prospective analysis from the Nurses’ Health Study found a twofold increase in risk of cholecystectomy with current MHT use, and this risk increased as duration of use increased^34^. A Danish population-based case–control study found an increased risk of gallstone disease in current and former estrogen users^35^. This study identified cases with gallbladder disease using diagnosis codes for gallstone disease or cholecystitis, and with procedure codes for gallbladder surgery^35^. The HERS-II trial found an increased risk of biliary tract surgery among women randomized to estrogen plus progestin^8^. This analysis included women with a history of gallbladder disease without cholecystectomy at baseline, and the outcome was defined as a hospital admission for acute cholecystitis, choledocholithiasis, or gallstone pancreatitis confirmed on abdominal ultrasonography or nuclear medicine scan^34^. Likewise, the WHI found significant increased risk of cholecystectomy among women in both the estrogen-only and estrogen plus progestin arms^7^. However, because silent gallstones represent 80% of all gallbladder disease, it is important to understand the risk of MHT use among all gallstone patients^1^.

A German population-based cross-sectional study found a significantly higher odds of cholecystectomy with ever use of MHT, but no association between MHT use and ultrasound-confirmed gallstones (both symptomatic and asymptomatic)^36^. Similarly, a Danish study that analyzed a cross-section of 2300 women from the National Person Register found no association between MHT use and gallbladder disease verified by abdominal ultrasonography^37^. The authors of these studies used traditional analysis methods, which may have biased their results due to confounding by contraindication. These studies also adjusted for several confounders (e.g., smoking, alcohol use, and oral contraceptive use) that were not significant in our models, which could explain the difference in results. Furthermore, two longitudinal studies found inverse associations between MHT use and gallbladder cancer^9,10^, likely due to confounding bias given the large body of evidence for gallstone formation with MHT use and the strong associations between gallstones and gallbladder cancer^1,38^.

To our knowledge, this is the first analysis to examine MHT use, and silent gallstones detected using abdominal ultrasonography, the diagnostic gold standard for gallbladder disease, and to control for cofounding by contraindication. However, this study has some limitations. We were unable to evaluate the odds of gallstones with different MHT formulations (e.g., estrogen-only or in combination with progesterone), as the NHANES III did not collect this information. Women in this analysis were surveyed between 1988 and 1994 and were likely to have been exposed to different formulations of MHT over time. For instance, pills containing conjugated equine estrogen only were commonly prescribed in the 1970s, while estrogen in combination with medroxyprogesterone pills was introduced in the 1980s^39^. Transdermal applications, which may confer a lower risk of gallstones by bypassing metabolism by the liver, were introduced in the 1980s^40^. Much of the clinical data on estrogen and gallbladder disease is based on studies using estrogen only formulations, and one study found that the use of unopposed estrogen was a greater risk factor for symptomatic gallstones than the use of opposed estrogen formulations^35^. IV analysis is a robust method that will produce unbiased estimates as long as the three key assumptions are met and the sample size is sufficient. While our instrument met the first two assumptions, it may not have been independent of some of the measured confounders. The IV analysis also resulted in large standard errors which reduced statistical power, diminishing our ability to detect significant differences between the traditional and IV analyses. With the goal of parsimony to reduce collinearity in the choice of confounders, we did not include some variables in our model, such as smoking, alcohol use, and oral contraceptive use, which could have resulted in residual confounding. Finally, because the NHANES is a cross-sectional survey, we cannot establish temporality, such that silent gallstones detected on examination may have been present before the initiation of MHT.

This analysis used the NHANES III, a survey conducted over 30 years ago. The prevalence of gallbladder disease may have changed since this study was conducted due to increasing trends in obesity among US adults. In 1980 the prevalence of obesity was 18% among males and 21% among females increasing to 32% among males and 34% among females in 2013^41^. There have been no more recent domestic prospective studies on gallbladder disease using the gold standard of abdominal ultrasound to understand the pathogenesis of gallstones. Our results underscore the need for ultrasound-based studies of gallstones; adding this examination to subsequent NHANES waves would be a useful first step. Furthermore, appropriate statistical methods that account for bias due to confounding by contraindication should be considered as the results may be misleading. Longitudinal studies of ultrasound confirmed gallstones are needed to verify our findings that MHT use is associated with an increase of asymptomatic gallstones.

### Supplementary Information

Supplementary Information.

## Data Availability

The data generated in this study are available from NHANES III for download: https://wwwn.cdc.gov/nchs/nhanes/nhanes3/datafiles.aspx.

## Abbreviations

2SRI: Two-stage residual inclusion
BMI: Body mass index
CI: Confidence interval
HERS-II: Heart and the estrogen/progestin replacement study
IV: Instrumental variable
MHT: Menopausal hormone therapy
NHANES III: Third National Health and Nutritional Examination Survey
OR: Odds ratio
SE: Standard error
US: United States
WHI: Women’s Health Initiative
