# Supplementary material for: Association with menopausal hormone therapy and asymptomatic gallstones in US women in the third National Health and Nutrition Examination Study
Source: Sci Rep. 2024 Jan 2;14:191. doi: 10.1038/s41598-023-50509-2 (PMC10761940; doi:10.1038/s41598-023-50509-2)
Supplement: Supplementary file 1 — Supplementary Information. [file 41598_2023_50509_MOESM1_ESM.docx]

**Supplementary Online Content**

SS Jackson, BI Graubard, C Gabbi, and J Koshiol. **Association with menopausal hormone therapy and asymptomatic gallstones in US women in the third National Health and Nutrition Examination Study (NHANES III).**

**eMethods**. Supplemental Methods

**eTable 1.** Associations between the instrument (geographic region) and the treatment (MHT use) for the three MHT models

**eTable 2.** Associations between the instrument (geographic region) and the outcome (asymptomatic gallstones) for the three MHT models

**eTable 3.** Balance of study baseline characteristics by the instrument (geographic region)

**eMethods**

**Instrumental Variable Selection**

The first step is to choose an instrument and verify that three key assumptions are met. These key assumptions are as follows: 1) the IV is associated with the treatment; 2) the IV affects the outcome only through the treatment; and 3) the IV is not associated with unmeasured confounders

after conditioning on measured confounders.^1^ These assumptions are illustrated in the **eFigure** below using our study’s exposure (MHT use), outcome (asymptomatic gallstones), and IV (geographic location).^2^ Following these assumptions, the instrument should have no direct or indirect effect on the outcome.^3^


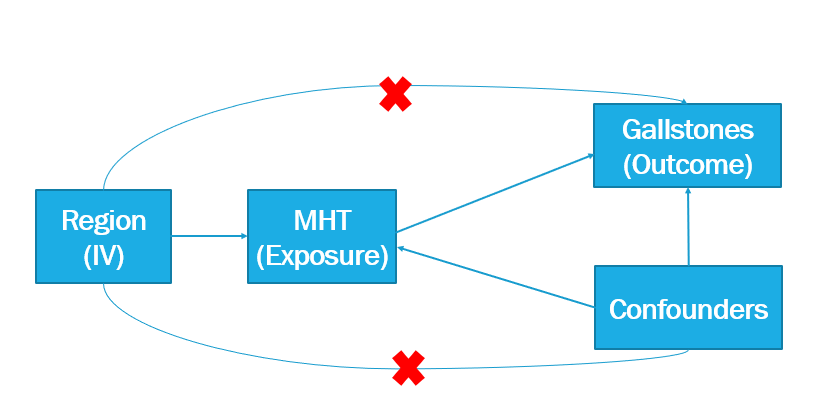


**eFigure**. A directed acyclic graph^2^ illustrating the relationships between the instrumental variable (geographic region), treatment exposure (MHT), unmeasured confounders, and the outcome (asymptomatic gallstones). The variable, geographic region, meets the assumptions of an instrument because it is associated with MHT use, has no direct effect on the outcome, and independent of unmeasured confounding conditional on covariates.

**Statistical Methods**

We next statistically tested these three key IV assumptions. We operationalized MHT use in three ways: 1) Current, former, or never MHT use; 2) Duration of use (0–<1 years, ≥1–<5 years, and ≥5 years); and 3) time since last use (0–<1 years, ≥1–<5 years, and ≥5 years). To test the first assumption, that the instrument is associated with treatment, we calculated the F-statistic with 1 degree of freedom for the IV in three separate regression models for MHT use as the dependent variables taking into account the complex survey design and clustering. An F-statistic less than 10 indicates a weak IV. As shown in **eTable 1**, our instrument, geographic location, meets the first assumption because the MHT use variables are strongly associated with geographic region and have F-statistics greater than 10.

| **eTable 1.** Associations between the instrument (geographic region) and the treatment (MHT use) for the three MHT models | | |
| --- | --- | --- |
| **MHT use** | **F- statistic** | ***P*-value** |
| Current, former, never | 27.82 | <0.0001 |
| Duration of use | 35.39 | <0.0001 |
| Time since last use | 13.73 | 0.0005 |

We next tested assumption 2, that IV affects the outcome only through the treatment. We examined the association between geographic region (Northeast, Midwest, South, and West) and asymptomatic gallstones in each MHT use model (current, former, never; duration; and time since last use) adjusting for age, race/ethnicity, body mass index category, parity, and type of menopause (natural, surgical/other) and taking into account the complex survey design and clustering. As shown in **eTable 2**, our instrument, geographic location, meets the second assumption geographic location is not associated with asymptomatic gallstones in any of the MHT use models.

| **eTable 2.** Associations between the instrument (geographic region) and the outcome (asymptomatic gallstones) for the three MHT models | | |
| --- | --- | --- |
| **MHT model** | **F- statistic** | ***P*-value** |
| Current, former, never | 0.52 | 0.67 |
| Duration of use | 0.53 | 0.66 |
| Time since last use | 0.51 | 0.68 |

Finally, we examined the assumption of the independence of the IV and unmeasured confounders. Though this assumption cannot tested completely using observed data we looked at the balance of measured covariates by geographic region. This approach is similar to examining the balance between baseline covariates across exposure categories in a conventional study.^1^ An imbalance in measured covariates suggests that there could also be unmeasured covariates associated with our instrument. We examined the distribution of important study characteristics among the four geographic regions taking into account the complex survey design and clustering. As shown in **eTable 3**, there were significant differences in women’s age at the time of survey, age at menopause, and type of menopause across the four geographic regions. This may mean that our assumption of independence of the IV is violated. This means that our instrument may not be strong and could lead to some bias in our results.

| **eTable 3.** Balance of baseline characteristics by the instrument (geographic region) | | | | | |
| --- | --- | --- | --- | --- | --- |
| **Characteristic** | **Northeast** | **Midwest** | **South** | **West** | ***P*-value**^a^ |
| Age in years, mean | 60 | 59 | 56 | 60 | 0.003 |
| Non-Hispanic White, % | 80 | 88 | 77 | 78 | 0.02 |
| Obese, % | 32 | 34 | 31 | 28 | 0.05 |
| Diabetes, % | 11 | 9 | 10 | 11 | 0.86 |
| Parity, mean | 2.9 | 2.9 | 2.8 | 3.0 | 0.51 |
| Age at menopause, mean | 45 | 43 | 41 | 44 | 0.003 |
| Natural menopause, % | 39 | 45 | 58 | 47 | <0.001 |
| ^a^*P*-values were estimated from an F-test for continuous variables and from a chi-square test for categorical variables. | | | | | |

**References**

1. Ertefaie A, Small DS, Flory JH, Hennessy S. A tutorial on the use of instrumental variables in pharmacoepidemiology. *Pharmacoepidemiology and drug safety.* 2017;26(4):357-367.

2. Baiocchi M, Cheng J, Small DS. Instrumental variable methods for causal inference. *Stat Med.* 2014;33(13):2297-2340.

3. Brookhart MA, Rassen JA, Schneeweiss S. Instrumental variable methods in comparative safety and effectiveness research. *Pharmacoepidemiology and drug safety.* 2010;19(6):537-554.
